# The Correction of Spinal Deformity by Manual Force

**Published:** 1898-04

**Authors:** 


THE CORRECTION OF SPINAL DEFORMITY BY MANUAL FORCE. —Dr. V. P. Gibney, of New York, one of the leaders in orthopedic surgery, has taken up the work, first promulgated by Calot and since performed by surgeons in England and on the continent, of correcting curvature of spine by manual force. Dr. Gibney is the surgeon-in-chief of the hospital for ruptured and crippled, and it was there that he carried out the suggestions of Calot, the first case being on the 1st of March, 1898. This case was that of a boy twelve years of age. The deformity was quite marked, and had existed as long as the boy could remember. The boss was in the lumbar region. He walked awkwardly, bending forward, and it was with difficulty that he could hold himself in the erect posture. All acute symptoms had long subsided, and inasmuch as the deformity occupied the region of the column in which the cauda equina is situated, Dr. Gibney could see no possible danger in forcible correction. On March 1st, under gas and ether, he had two assistants to take the lower limbs, two the upper, and, with a hip rest under the thighs and a broad one under the upper part of the thorax, he employed manual force over the boss. The doctor could distinctly feel the parts yielding, with a noise which was heard over the operating room. This part of the operation was completed within a few minutes. The parts where pressure had been made were then padded with piano felting, also the iliac crests. A close-fitting plaster of Paris jacket was then applied while the patient was in the recumbent posture. The patient rallied quickly from the anesthetic, and had no elevation of temperature nor any untoward symptom. On the third day he was allowed to walk about, which he did perfectly erect.

The next case was that of a boy six and a half years old. His deformity was mid-dorsal, and had existed about three years, at the time of the operation, March 8th. The procedure was identical with the first case, and with the same good results. On the third day the patient was walking around, and had not suffered any pain.

The third case was that of a girl fourteen years of age. Her deformity had existed about five years. The patient had a typical hunchback. After being placed under gas and ether, she was laid on her left side with a hip rest under the hip, one in the axillary space, and, while traction was being made by two assistants, Dr. Gibney forced the column into a much better position. The deformity in this case was not fully corrected, but there was at least an inch gain in height. Plaster of Paris was applied as in the other cases. No ill results followed the operation. The procedure is to be repeated in the course of a week or so, with the confident hope that the deformity will be overcome.

These cases represent three types of spinal deformity, which heretofore had not been improved by any treatment in vogue. The various braces at best gave only freedom from pain without special improvement of the deformity. It is only a wonder that this simple procedure has not been thought of long ago. It marks an advance in the treatment of this common deformity.

---

"The Bovinine Company's contracts were sent out, this year, through Mr. H. G. Elliott, a New York advertising agent. In many instances, I understand, the rates attached were not at all satisfactory to the publishers receiving them. As Mr. Elliott is decidedly new in the medical journal line, the question naturally presents itself: Where did he get his very insignificant idea of the value of space in medical magazines? It is unfortunate, in my opinion, and surprising, as well, that so shrewd and capable a manager as Mr. Champney, should have allowed his intimacy with the editorial guild to have been disturbed by the introduction of a newspaper advertising agent, with his insufferable methods. A little time, and perhaps some money, may be saved to the Bovinine Company, but will it equal what is lost in "entente cordiale?"—*American Medical Journalist.*

We think not. Speaking for ourselves, our business relations with Mr. Champney have heen very pleasant indeed, in fact cordial. We regret exceedingly to have them interrupted in any way, but this new man saw fit to set such a price on our space that we were forced, in order to preserve our self-respect, to decline it, and to volunteer the suggestion to Mr Elliott that if he will permit us to do so, we prefer to name the price on our own space. In the main our business with advertising agents has been pleasant and we have felt that we could work together for mutual good, but such a fellow as this gives us "that tired feeling."

---

THE AMERICAN MEDICAL ASSOCIATION MEETING in Denver, June 7, 8, 9 and 10. This bids fair to be one of the largest meetings this great Association with its many thousands of members, has ever held. We hope for and expect a large attendance from Texas. There is so much to attract the Texas doctor to Colorado. It is the best summer resort for our pleasure and health-seeking people; so many of our people, especially those suffering from lung diseases, have been restored to health by a residence in that magnificent climate. There are very many more who would be greatly benefited by going there, and they would gladly make the trip if they knew how much their health would be improved by it. Their physicians express some doubt about it, or do not even suggest the matter at all, because they do not feel quite sure that the climate is so perfect or the air so pure as it has been represented to them. They would gladly send their patients anywhere if they could have the assurance that they would be restored to health. Personal inspection and observation will fully convince the most skeptical. This is an excellent opportunity for the Texas doctors to visit Colorado and determine for themselves whether it has the grand scenery and the pure health-giving atmosphere that has been reported of it. The trip will be a most enjoyable one, and with the many attractions furnished by the country, the city of Denver and the meeting itself, we feel safe in offering to guarantee every delegate from Texas a royal good time and the full worth of the time and money spent.

